# 

**Published:** 1898-01-15

**Authors:** 


**Notices of Births, Deaths, and Marriages** are inserted in this column at a charge of 2s. 6d. for an announcement not exceeding four lines.

### NOTICE TO CORRESPONDENTS.

All MS., Letters, Books for Review, and other matters intended for the Editor should be addressed The Editor, "The Hospital," The Hospital Building, 28 & 29, Southampton Street, Strand, W.C.

The Editor cannot undertake to return rejected MS., even when accompanied by stamped directed envelope.

**Notice to Advertisers and Subscribers.**

All Advertisements, Orders for Copies of The Hospital, and Business Communications should be addressed to **THE MANAGER, (Not to The Editor),** The Hospital, The Hospital Building, 28 & 29, Southampton Street, Strand, London, W.C.

The Cost of Subscription, post free, is as follows:—Per week, 2½d. per quarter, 2s. 8d.; per half-year, 5s. 3d.; per annum, 10s. 6d. Foreign:—Per annum, 15s. 2d.: payable, in all cases, in advance.

**NOTICE.—Vol. XXII. of "The Hospital" (April, 1897, to September, 1897), in handsome cloth binding, is now on sale at the Office of this Journal, price 7s. 6d. Cases for binding the Numbers contained in this Volume, 1s. 6d. Index to Vol. XXII., 2½d., post free.**

**The Monthly Part for January is now ready. Price 6d., by post 8d.**

### BOOKS RECEIVED.

SWAN SONNENSCHEIN AND CO.

"Introduction to the Study of Organic Chemistry." By John Wade, B.Sc.,Lond.

ISBISTER AND CO.

"Lincoln Cathedral." By the Rev. Edmund Venables, M.A. Illustrated by Herbert Railton.

BRITISH MEDICAL ASSOCIATION.

"Supplementary Croonian Lecture." By F. W. Pavy, M.D., F.R.S.

WARD, LOCK, AND CO.

"The Poultry Book."

**Periodicals Received.**—Boy's Own Paper, Girl's Own Paper, African Critic, Herald of Health, Sunday at Home, Charity Organisation Review, The Melbourne Sun New Age, Woman's Signal, Local Government Journal, Humanitarian, Scalpel, Epicure, Medical Pioneer.

### SCRAPS AND GLEANINGS.

THE Fleet Cottage Hospital has been opened.

THE foundation-stone of the Chippenham Cottage Hospital has been laid.

THE Lincoln Hospital Saturday Fund since its establishment in 1879 has produced nearly £11,000.

A VERY complete new operating department has recently been opened at the Leeds General Infirmary.

DURING the year 1896 7, 47 in and 33 out-patients received the benefits of the Driffield Cottage Hospital.

DURING the year ended August, 1897, 58 patients were treated at the Huntly Jubilee Cottage Hospital.

THE Grantham Hospital House to House Committee handed £200 over to the Grantham Hospital in 1897.

IT is expected that the new Stranraer Cottage Hospital will be opened in the spring or early summer of this year.

THE number of women admitted to the Cork Lying-in Hospital during the year ended October 31st, 1897, was 258.

THE total receipts of the Lincoln Hospital Saturday Fund in 1897 were £34', of which £308 was paid in to the hospital.

THE Queen has forwarded a gift of pheasants for the use of the Patients of the Cancer Hospital (Free) Fulham Road, S.W.

A COTTAGE hospital has been established at Torrington, Devonshire.

FORTY-TWO in-patients were admitted to the Axminster Cottage Hospital in 1896-7, the same number as in 1895-6.

THE number of patients treated last year at the Dudley Dispensary was 4,852, the smallest number received since 1886.

LAST year's collection at Burton-on-Trent in connection with Infirmary Saturday was again a record one, viz., £1,178.

OF the 146 cases treated at the Dublin Orthopædic Hospital last year 92 were cured. One thousand three hundred patients attended at the dispensary.

THE Duke of Westminster last year forwarded to the Chester General Infirmary £500 out of the moneys received from visitors viewing the Hall and gardens at Eaton.

THE Saturday night subscriptions from working men towards the maintenance of the Cardiff Infirmary was £20 less in 1896 than it was in 1886 (£770).

COVENTRY Hospital Saturday collection last year totalled £1,4[illegible]0, making £16,64[illegible] subscribed by the fund to the Coventry and Warwickshire Hospital since 1874.

AT the Dumbarton Public Eye Hospital 832 patients were treated during the year ended September 30th, 1897. The income amounted to £19[illegible], and the expenses to £191.

### THE STUDENT OF MEDICINE.

### APPOINTMENTS.

J. P. Bush, M.R.C.S.Eng., L.S.A., appointed Senior Surgeon to the Bristol Police Force. E. Carbery, M.B., B.Ch, B.A.O.-Dub., appointed House Physician to the City of London Hospital for Diseases of the Chest, Victoria Park. C. Challinor, M.R.C.S.-Eng., L.R.C.P.Lond., D.P.H.Vict., appointed Certifying Factory Surgeon for Leigh (Lancashire) District. G. Dickson, L.R.C.P.E., L.R.C.S E., L.F.P.S.Glasg., appointed Medical Officer of Health by the Rural District Councils of Hay, Breconshire; Painscastle, Radnorshire; and Bredwardine, Herefordshire. J. D. Doherty, M.B., M.S., appointed Assistant House Surgeon to the Northern Hospital, Liverpool. A. Gardner, M.B., C.M Edin., appointed Medical Officer of Health to the Kirby Moorside Rural District Council. T. A. Green, M.D, C.M.Edin., appointed Medical Officer of Health by the Meltham Urban District Council. S. Hamilton, B.A., M.B., B.Ch., appointed Medical Officer and Public Vaccinator to the Saint Mellous District of the Newport, Monmouthshire Union. W. Herbert, M.R.C.S., L.R.C.P., appointed Resident Medical Officer to the Wirral Children's Hospital, Birkenhead. T. Homer, L.R.C.S., L.R.C.P.-Edin., appointed Medical Officer of Health for the Small Isles District of Inverness-shire, and Parochial Medical Officer and Public Vaccinator for Small Isles, Inverness shire. E. H. Houfton, M.B.Lond., M.R.C.S.Eng., appointed Medical Officer of Health to the Mansfield Workhouse Urban District Council. A. Leche, L.R.C.P Edin.,M.R.C.S.Eng, appointed Medical Officer to the Workhouse and to the No. 7 District of the Axbridge Union. L. J. Lloyd, M.R.C.S., L.R.C.P., appointed Assistant Medical Officer of the St. Mary Abbotts (Kensington) Workhouse Infirmary. J. Mandall Coates, M.B., M.S.Edin., appointed House Physician to the Northern House, Liverpool. S. Moore, M.D., M.Ch., M.A.O., Roy Univ. Irel., appointed Medical Officer to the West District of the Holbeck Union. C. E. Paterson, M.D., appointed Medical Officer for the Frimley District of the Farnham Union. F. H. Pearce, B.A.Camb., M.R.C.S.Eng., L.R.C.P., appointed House Surgeon to Northern Hospital, Liverpool. C. E. Wallis, M.R.C.S., L.R.C.P., L.D.S., appointed Dental Surgeon to the Victoria Hospital, Chelsea.

### NATIONAL HOSPITAL FOR THE PARALYSED AND EPILEPTIC.

The following lectures will be delivered on Tuesdays at 3.30 p.m.: Jan. 11th, Dr. Beevor, Cerebral Localisation; Jan. 18th, Sir W. Gowers, F.R.S.; Jan. 25th, Dr. Ferrier, F.R.S.; Feb. 1st, Dr. Ormerod; Feb. 8th, Dr. Buzzard; Feb. 15th, Dr. Tooth, Cranial Nerves; Feb. 22nd, Sir F. Semon, Laryngeal Neuroses of Cortical Origin; March 1st, Mr. Victor Horsley, F.R.S., Surgery of the Nervous System; March 8th, Mr. Ballance, Surgery of the Nervous System; March 15th, Dr. Taylor; March 22nd, Mr. Gunn, Optic Atrophy.

